# Supplementary material for: Russian forests show strong potential for young forest growth
Source: Commun Earth Environ. 2025 Jan 30;6(1):71. doi: 10.1038/s43247-025-02006-9 (PMC11782080; doi:10.1038/s43247-025-02006-9)
Supplement: Supplementary file 1 — Supplementary Materials [file 43247_2025_2006_MOESM1_ESM.pdf]

Supplementary Materials for

Russian forests show strong potential for young forest growth

Christopher S.R. Neigh *et al.*
*Corresponding author. Email: christopher.s.neigh@nasa.gov

This PDF file includes:

Supplementary Text
Supplementary Figures 1 to 18
Supplementary Table 1

Histograms and maps of age, disturbed height and undisturbed height are provided in figures 1-5.

Maps of site index and errors and per-pixel *space-for-time* substitution samples of Site Index (SI) curves from Landsat stand age (x-domain) and ICESat-2 ATL08 hcan stand height (y-domain), for 10 of 527 available latitude plot transects organized by 0.5° longitude increments are provided in figures 6-15.

Histograms of the growth gap, change hotspots, and growth gap hotspots by country are provided in figure 16.

Maps of Landsat scene counts and the process for estimating tree canopy cover, forest probability, change and age are provided in figures 17 and 18.

Formulation of forest growth models is provided in Supplementary Table 1.

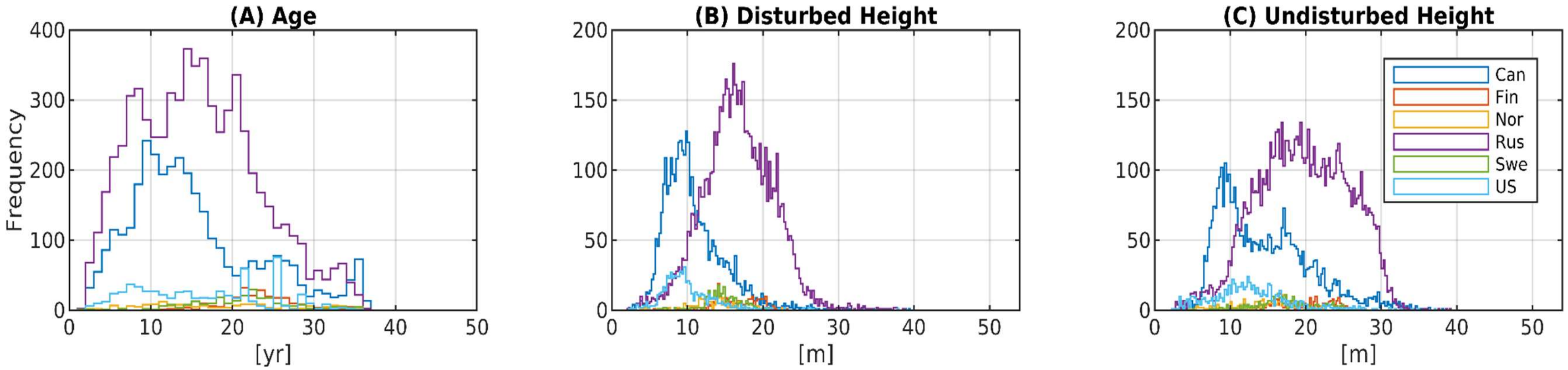

Supplementary Figure 1. Histograms of age **(A)**, disturbed height **(B)**, and undisturbed height **(C)** by country.

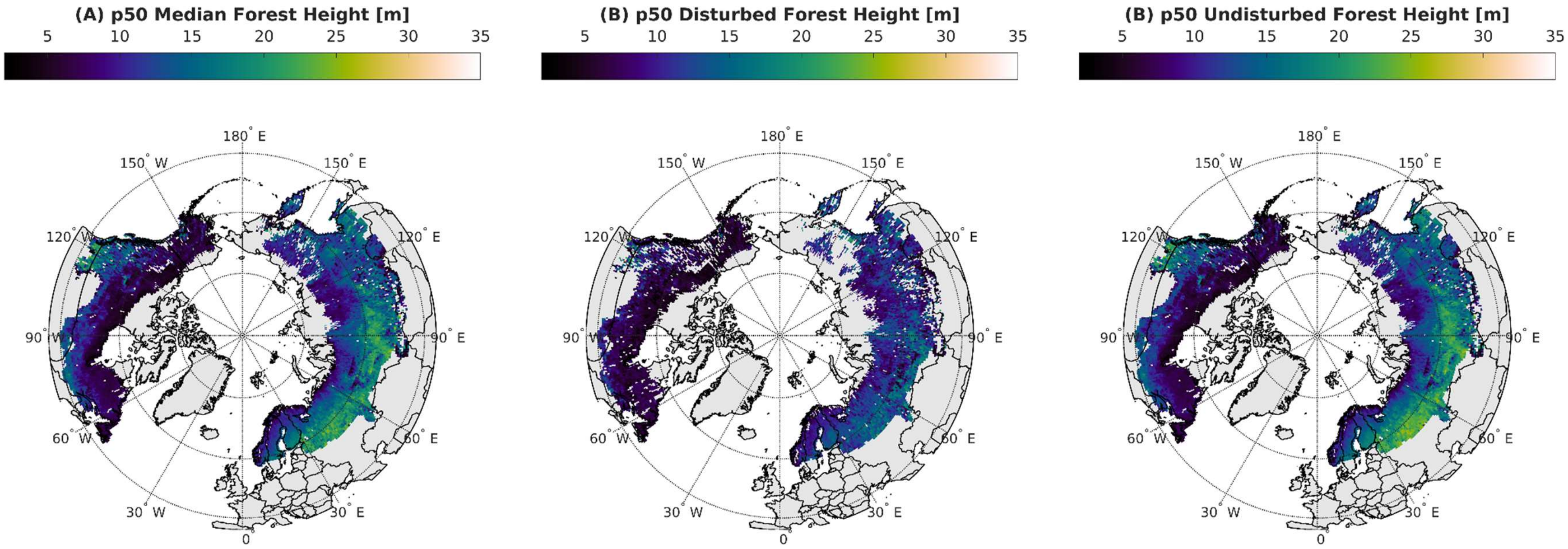

Supplementary Figure 2. ICESat-2 hcan 20 m geo-segment height median values at 0.5° × 0.5° for **(A)** all the boreal forest, **(B)** undisturbed, and **(C)** disturbed.

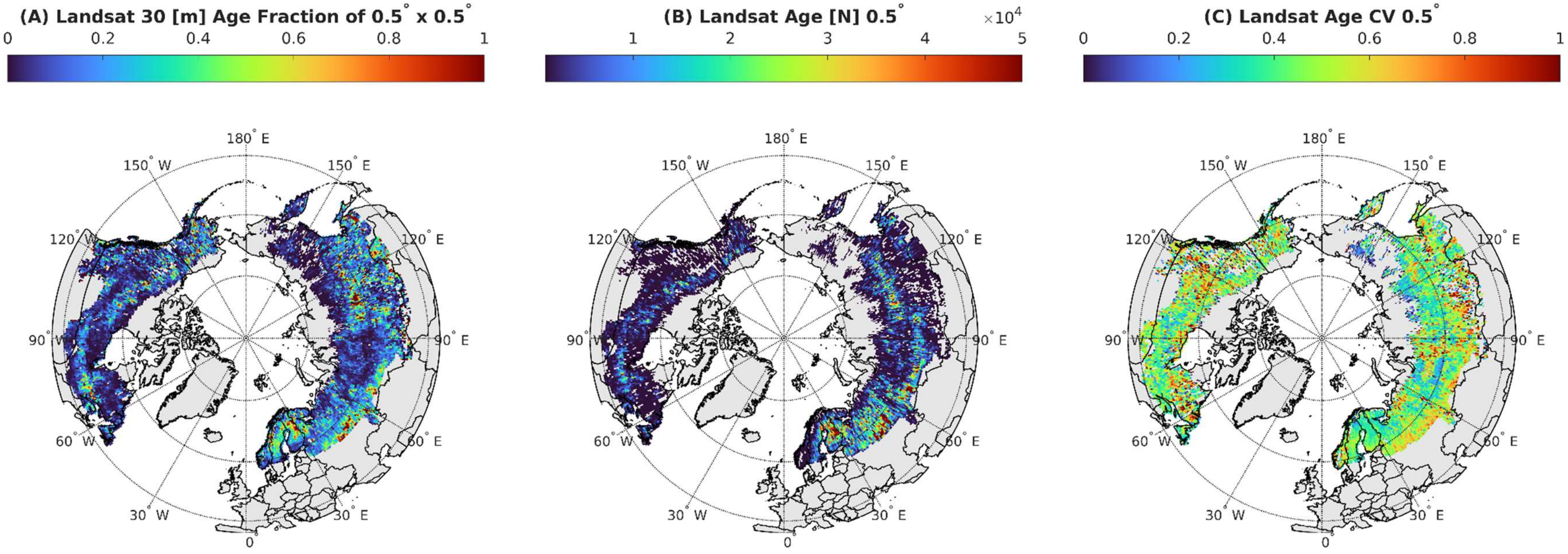

Supplementary Figure 3. (A) Landsat 30 [m] 1984 to 2020 age fraction of 0.5° × 0.5° grid, **(B)** number of disturbed samples, and **(C)** Landsat age coefficient of variation.

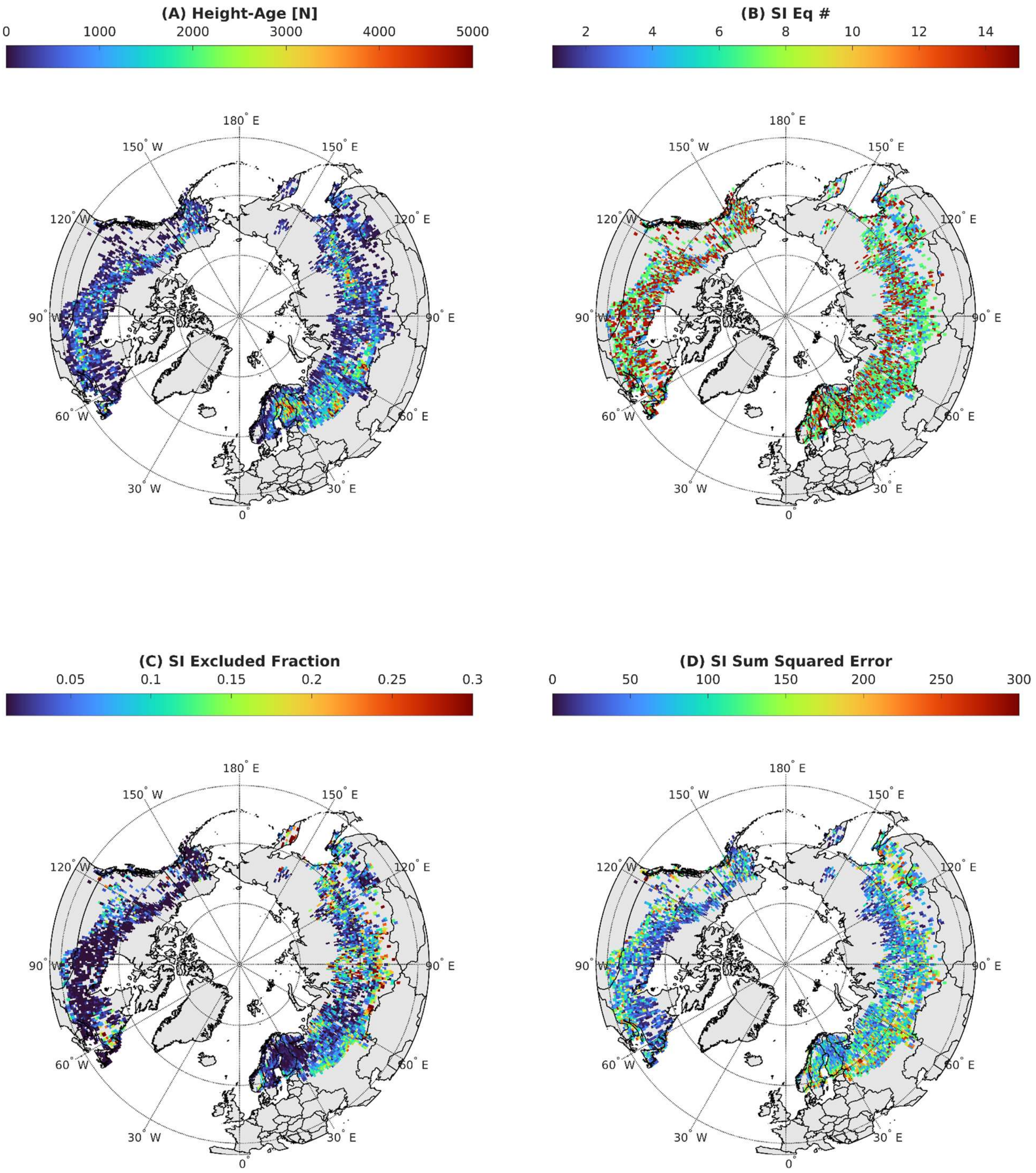

Supplementary Figure 4. (A) Number of Landsat age and ICESat-2 segment samples resolved at a 0.5° × 0.5° grid. **(B)** Site Index (SI) growth model applied from supplement table 1., **(C)** fraction of samples excluded in growth formulation, and **(D)** SI sum squared error.

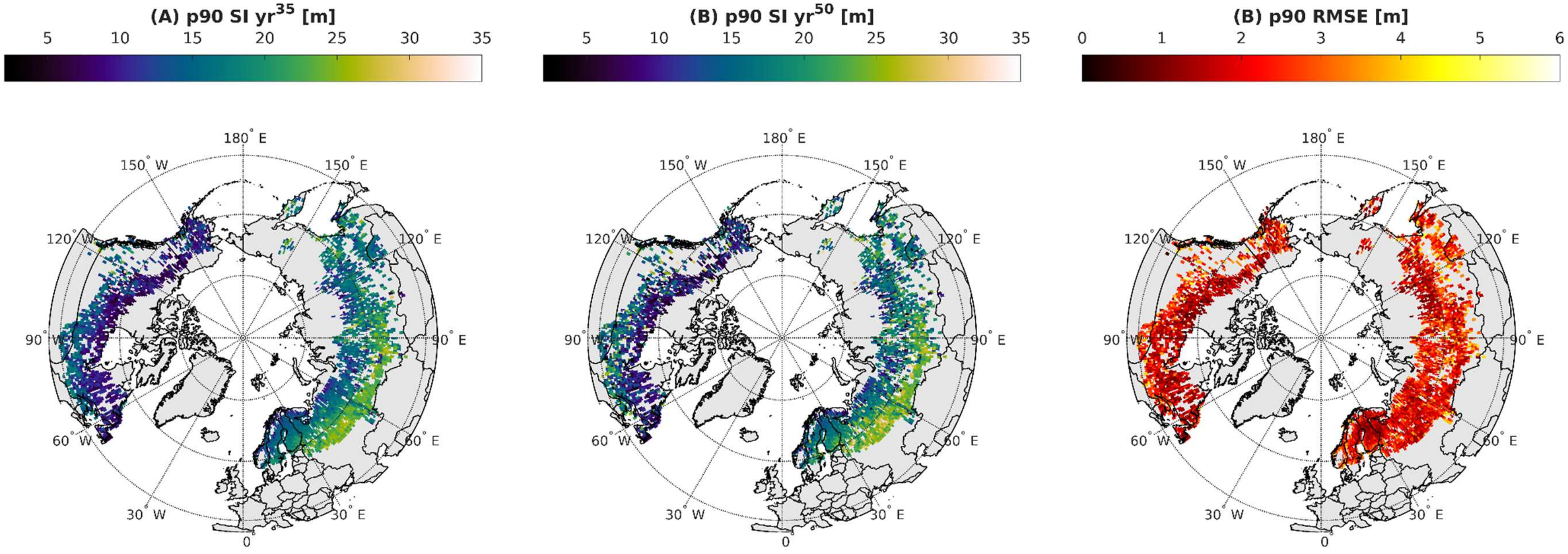

Supplementary Figure 5. Site Index (SI) calculated at 0.5° × 0.5° from the 90th percentile for year 35 **(A)** and year 50 **(B)** for all the boreal forest, and the root mean square error (RMSE) of the selected growth model **(C)**.

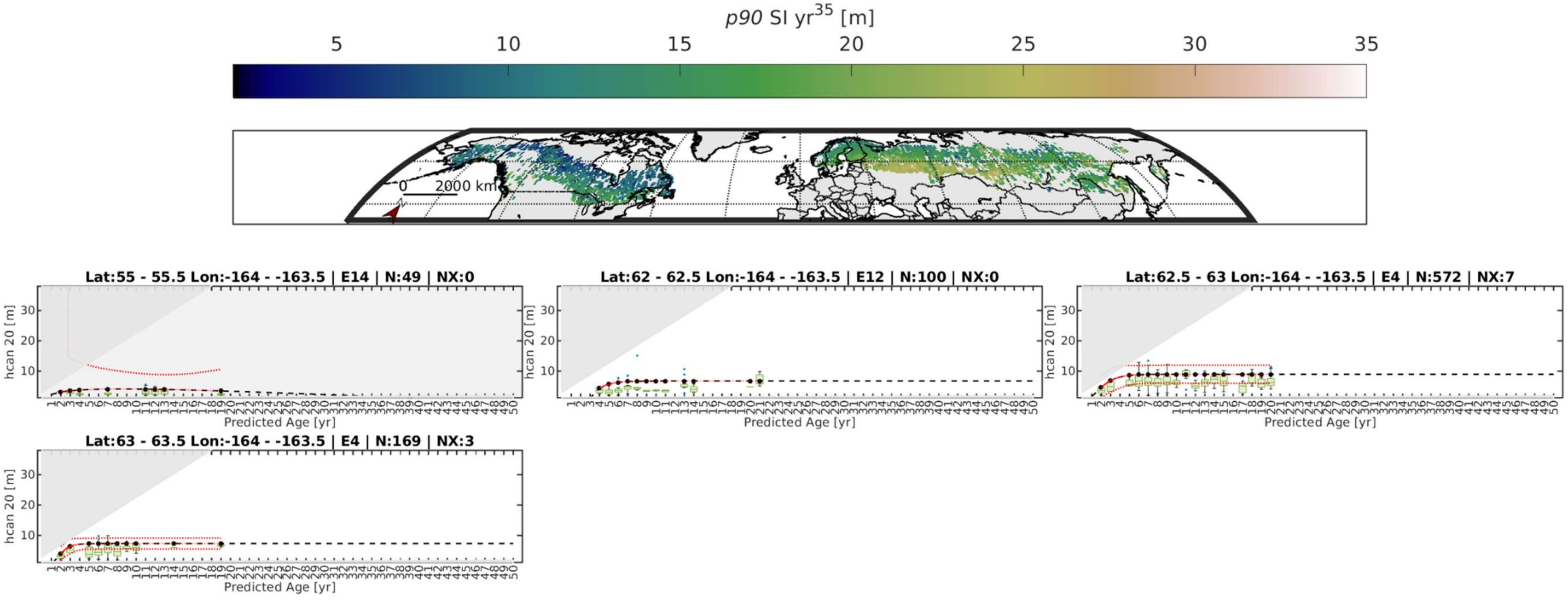

Supplementary Figure 6. Per-pixel latitude transects from -164° to -163.5° longitude indicated by the red North arrow of SI year 35 curves. Red dashed lines indicate 95% confidence bounds when calculation was possible. Dark gray plots did not meet RMSE and SSE thresholds and were excluded from SI analysis.

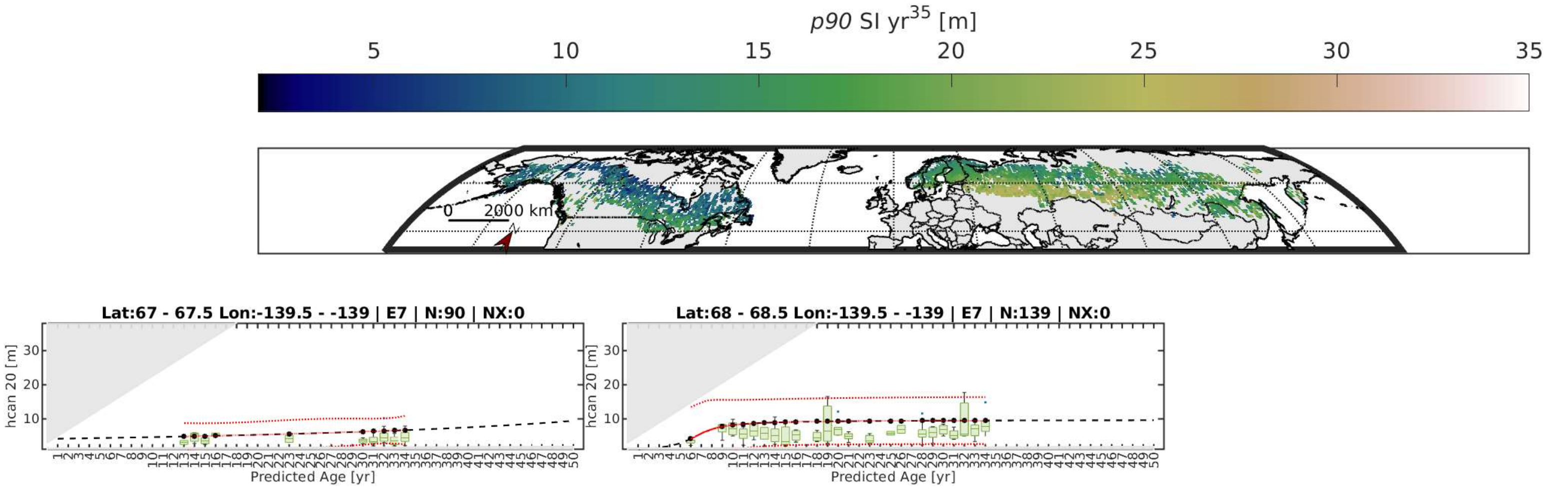

Supplementary Figure 7. Per-pixel latitude transects from -139.5° to -139° longitude indicated by the red North arrow of SI year 35 curves. Red dashed lines indicate 95% confidence bounds.

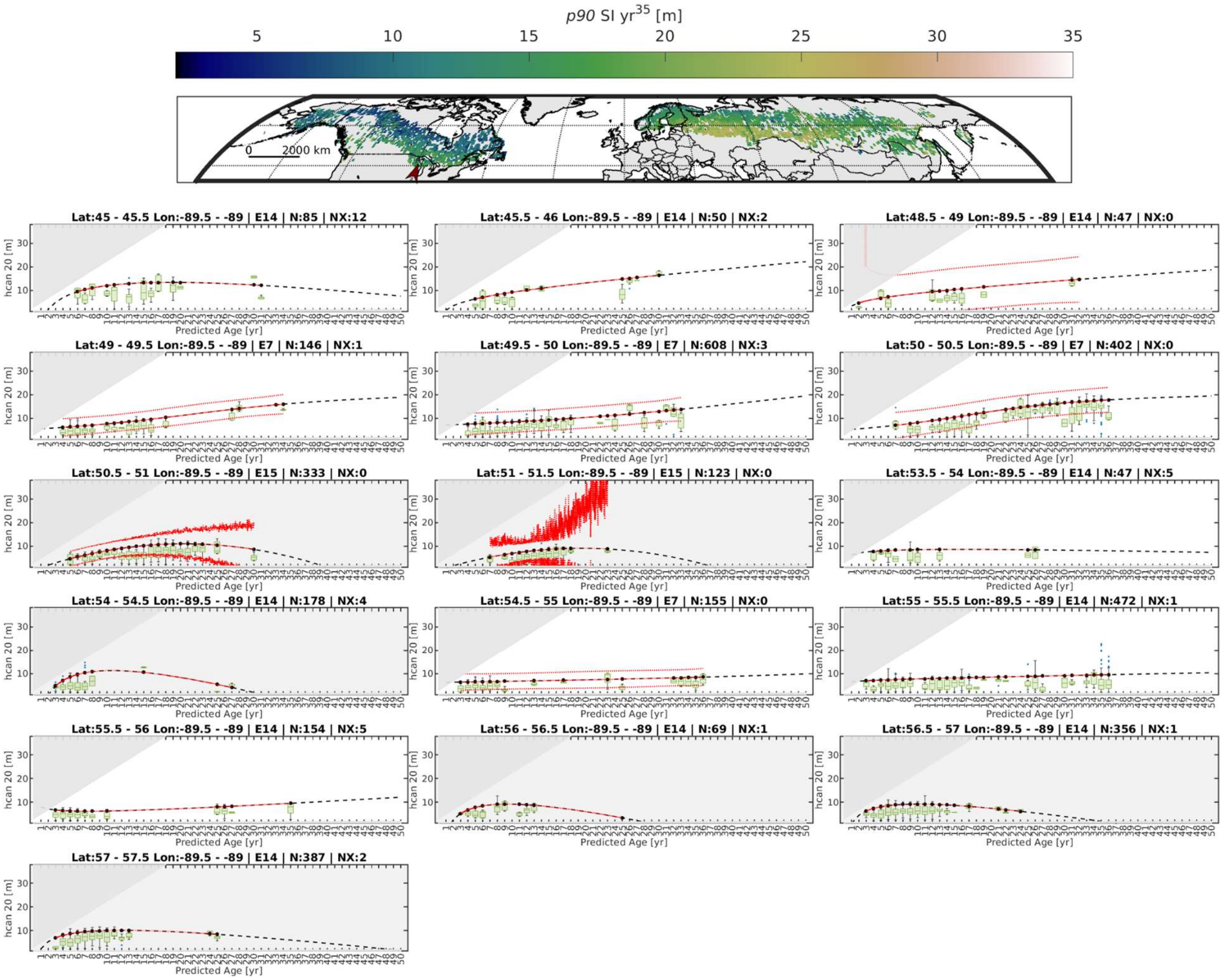

Supplementary Figure 8. Per-pixel latitude transects from -89.5° to -89° longitude indicated by the red North arrow of SI year 35 curves. Red dashed lines indicate 95% confidence bounds when calculation was possible. Dark gray plots did not meet RMSE and SSE thresholds and were excluded from SI analysis.

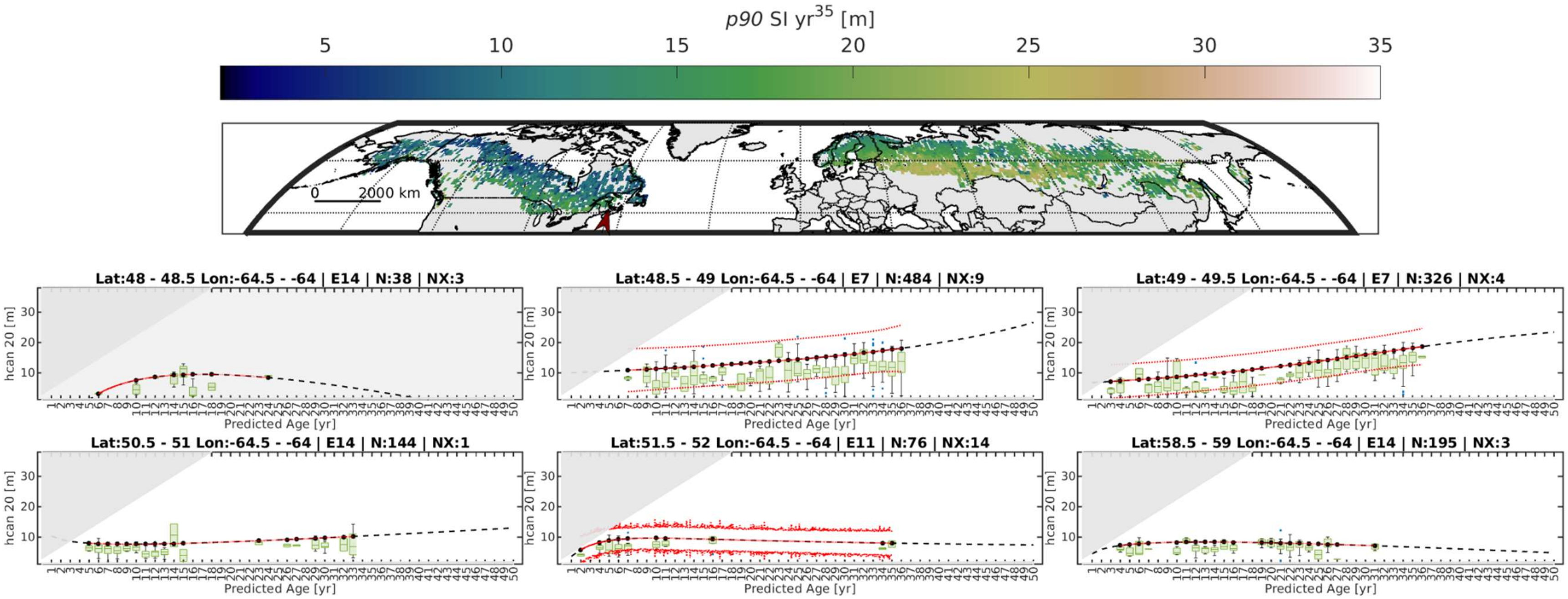

Supplementary Figure 9. Per-pixel latitude transects from -64.5° to -64° longitude indicated by the red North arrow of SI year 35 curves. Red dashed lines indicate 95% confidence bounds when calculation was possible. Dark gray plots did not meet RMSE and SSE thresholds and were excluded from SI analysis.

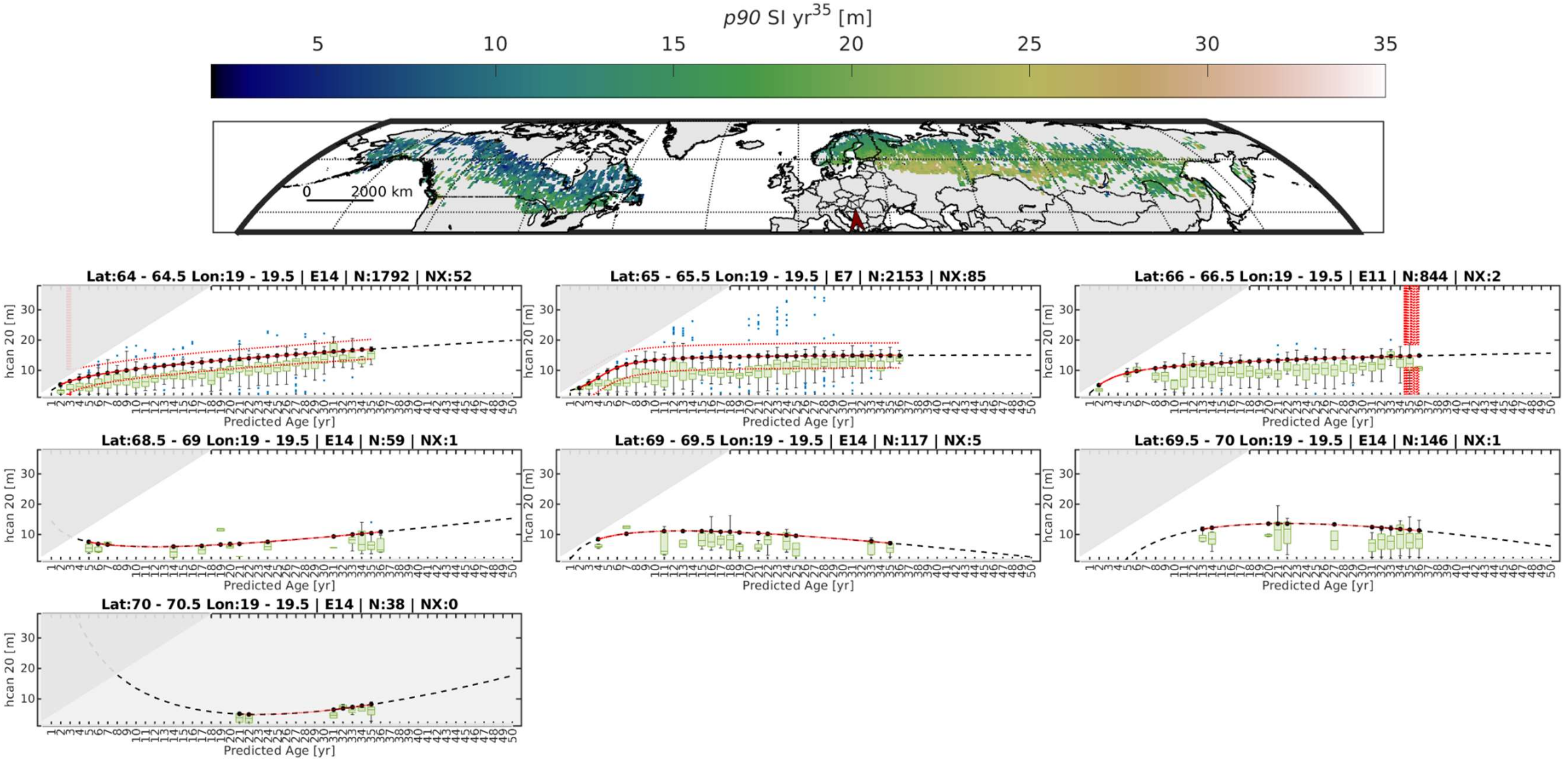

Supplementary Figure 10. Per-pixel latitude transects from 19° to 19.5° longitude indicated by the red North arrow of SI year 35 curves. Red dashed lines indicate 95% confidence bounds when calculation was possible. Dark gray plots did not meet RMSE and SSE thresholds and were excluded from SI analysis.

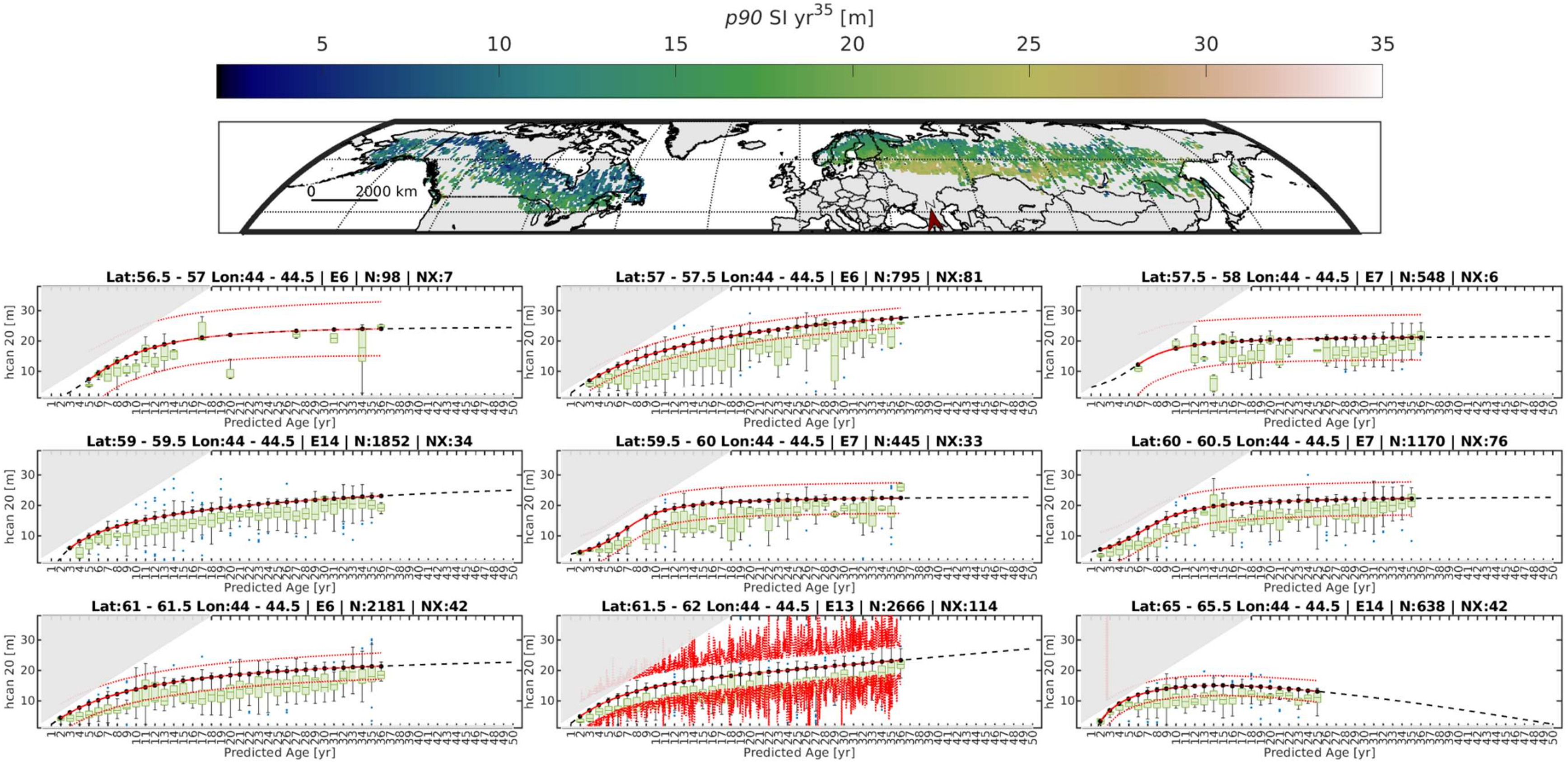

Supplementary Figure 11. Per-pixel latitude transects from 44° to 44.5° longitude indicated by the red North arrow of SI year 35 curves. Red dashed lines indicate 95% confidence bounds when calculation was possible.

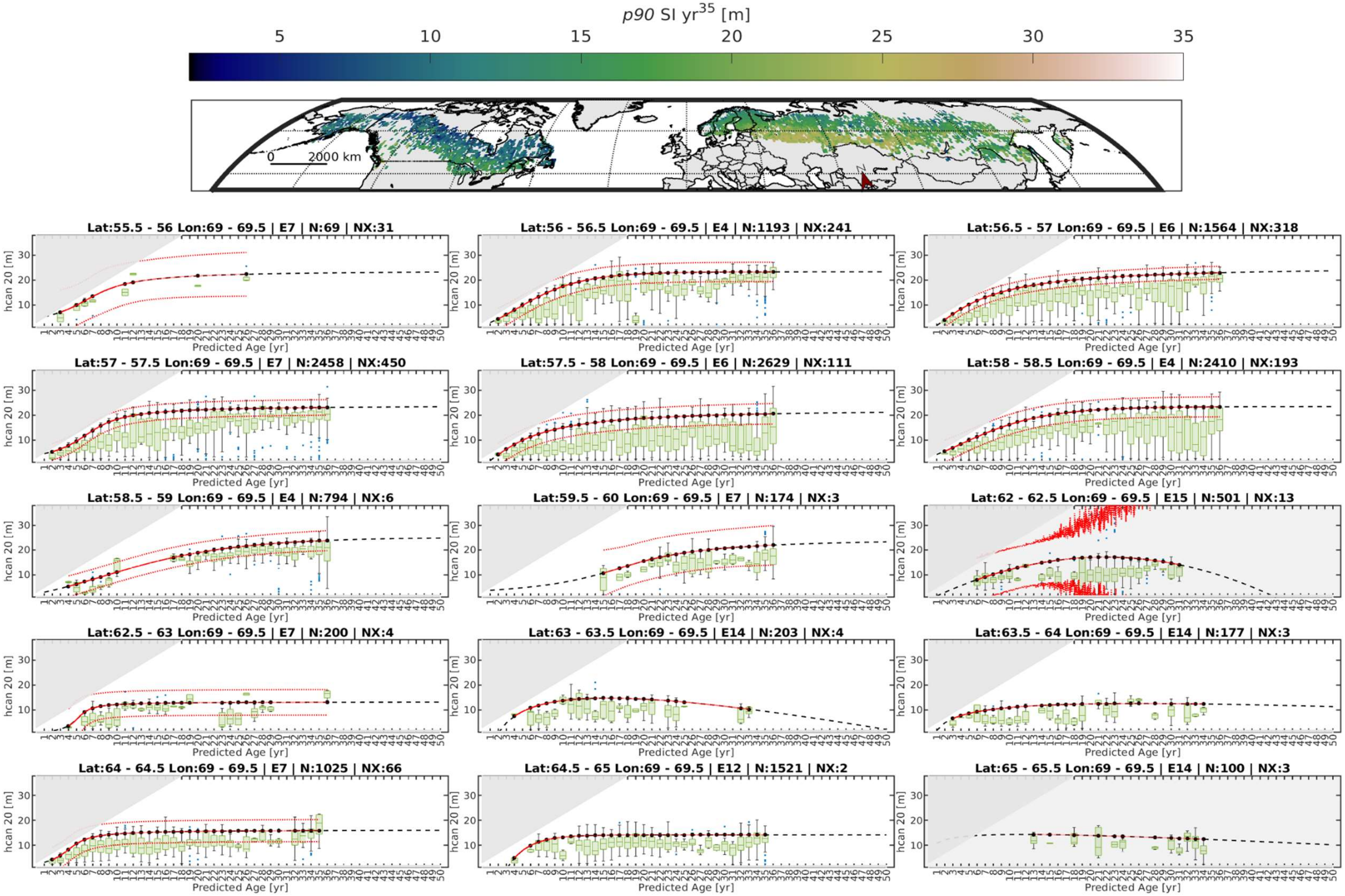

Supplementary Figure 12. Per-pixel latitude transects from 69° to 69.5° longitude indicated by the red North arrow of SI year 35 curves. Red dashed lines indicate 95% confidence bounds when calculation was possible. Dark gray plots did not meet RMSE and SSE thresholds and were excluded from SI analysis.

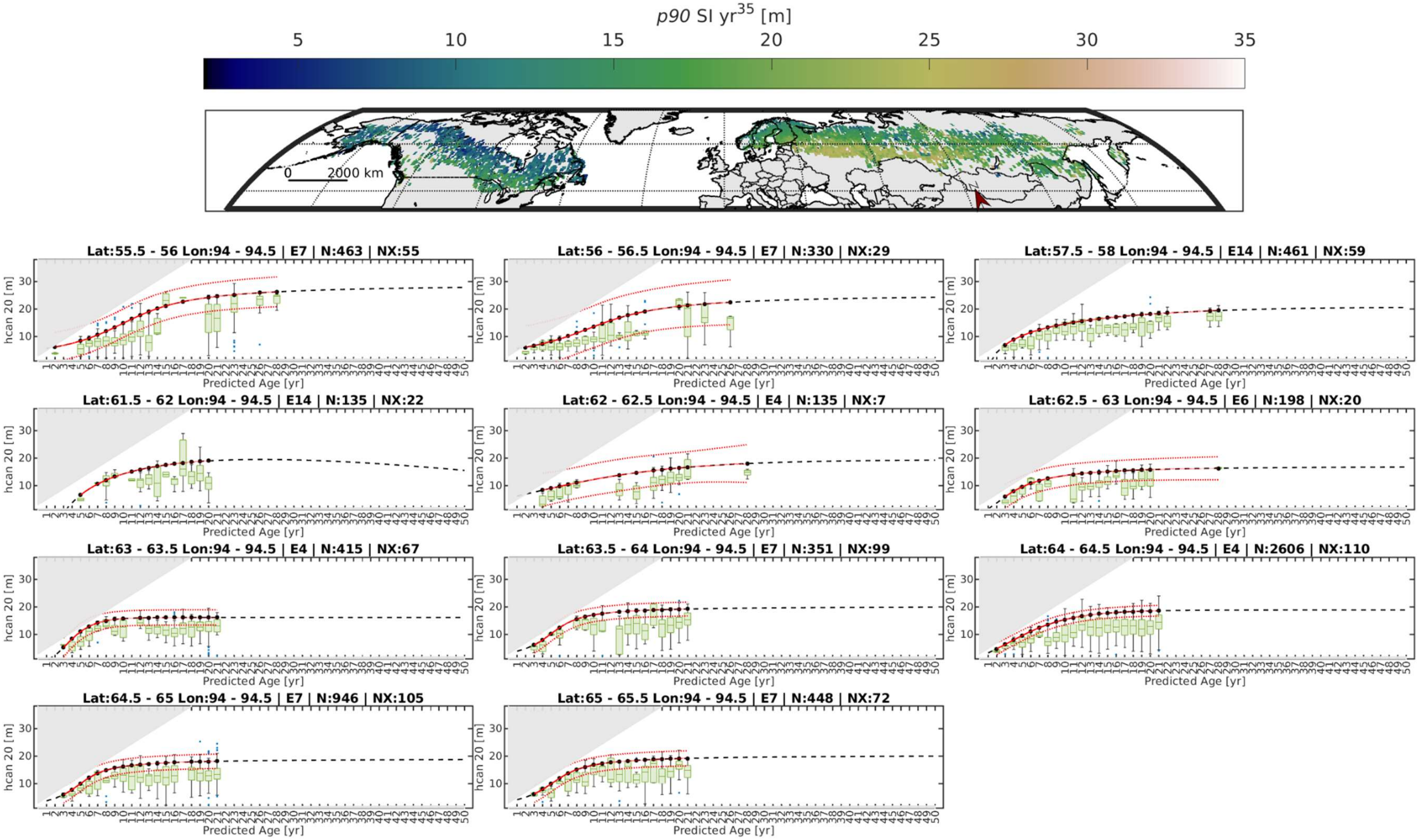

Supplementary Figure 13. Per-pixel latitude transects from 94° to 94.5° longitude indicated by the red North arrow of SI year 35 curves. Red dashed lines indicate 95% confidence bounds when calculation was possible.

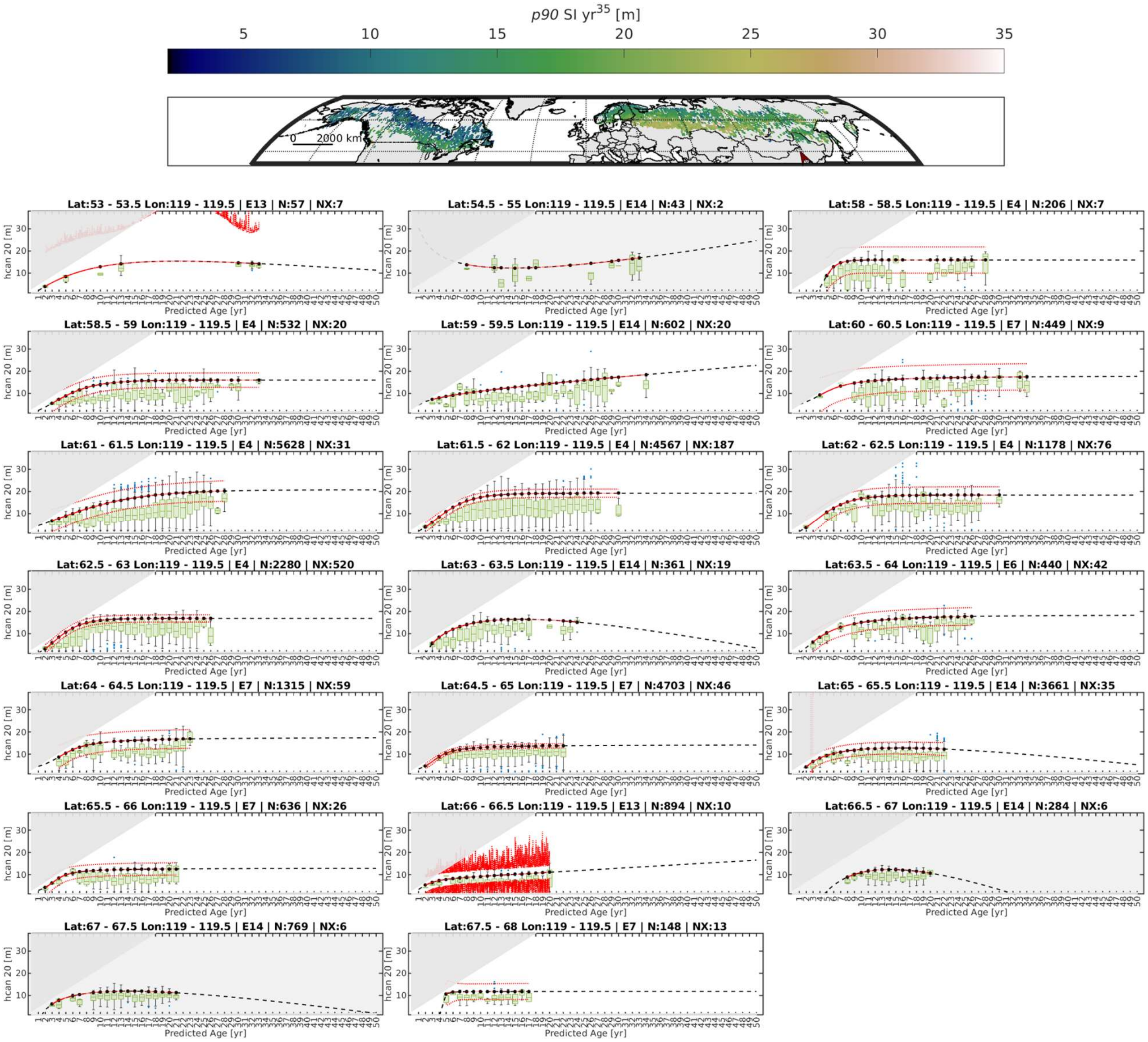

Supplementary Figure 14. Per-pixel latitude transects from 119° to 119.5° longitude indicated by the red North arrow of SI year 35 curves. Red dashed lines indicate 95% confidence bounds when calculation was possible. Dark gray plots did not meet RMSE and SSE thresholds and were excluded from SI analysis.

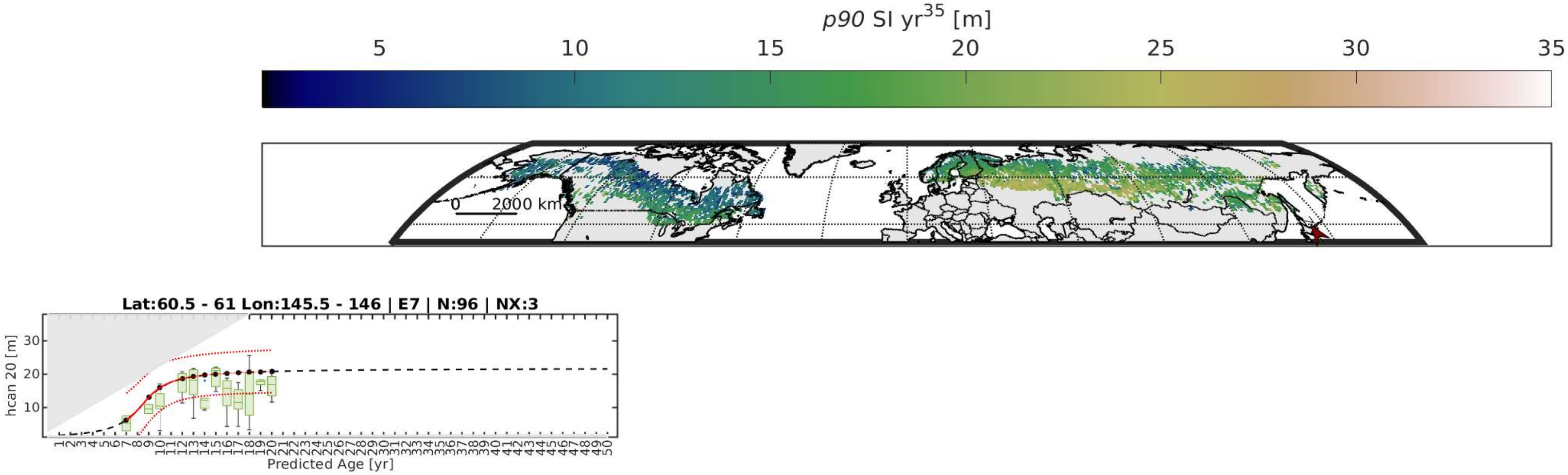

Supplementary Figure 15. Per-pixel latitude transects from 145.5° to 146° longitude indicated by the red North arrow of SI year 35 curves. Red dashed lines indicate 95% confidence bounds when calculation was possible.

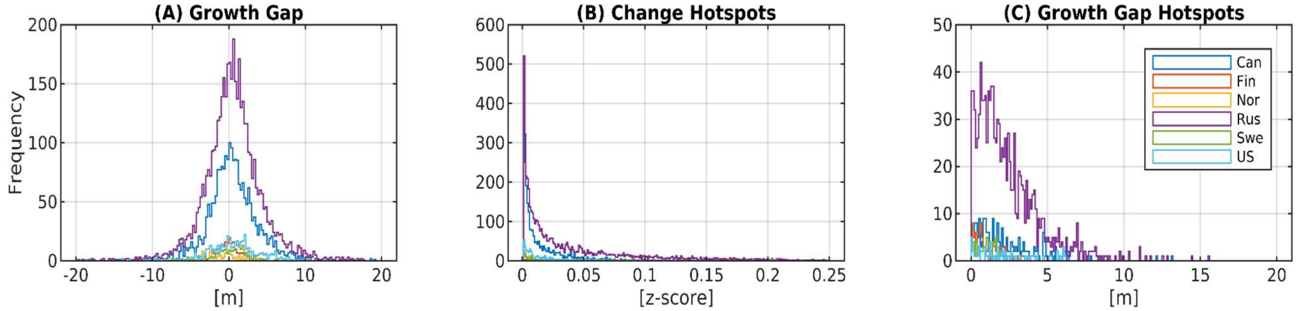

Supplementary Figure 16. Histograms of the growth gap **(A)**, change hotspots **(B)**, and growth gap hotspots **(C)**. Growth gaps defined as predicted minus observed, change hotspots defined as greater than the third quartile of the z-score of disturbance per gridcell, and growth gap hotspots are the combination of the growth gap and change hotspots.

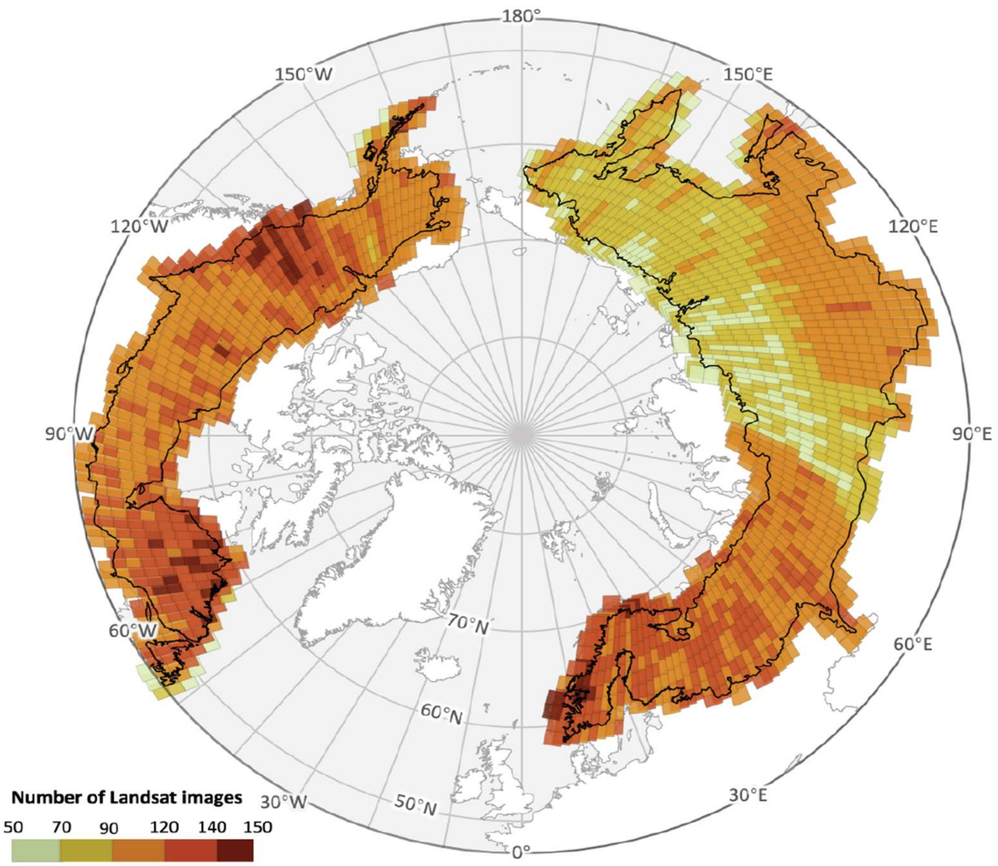

Supplementary Figure 17. Spatial distribution of sampling density of Landsat images across the study region from 1984 - 2020.

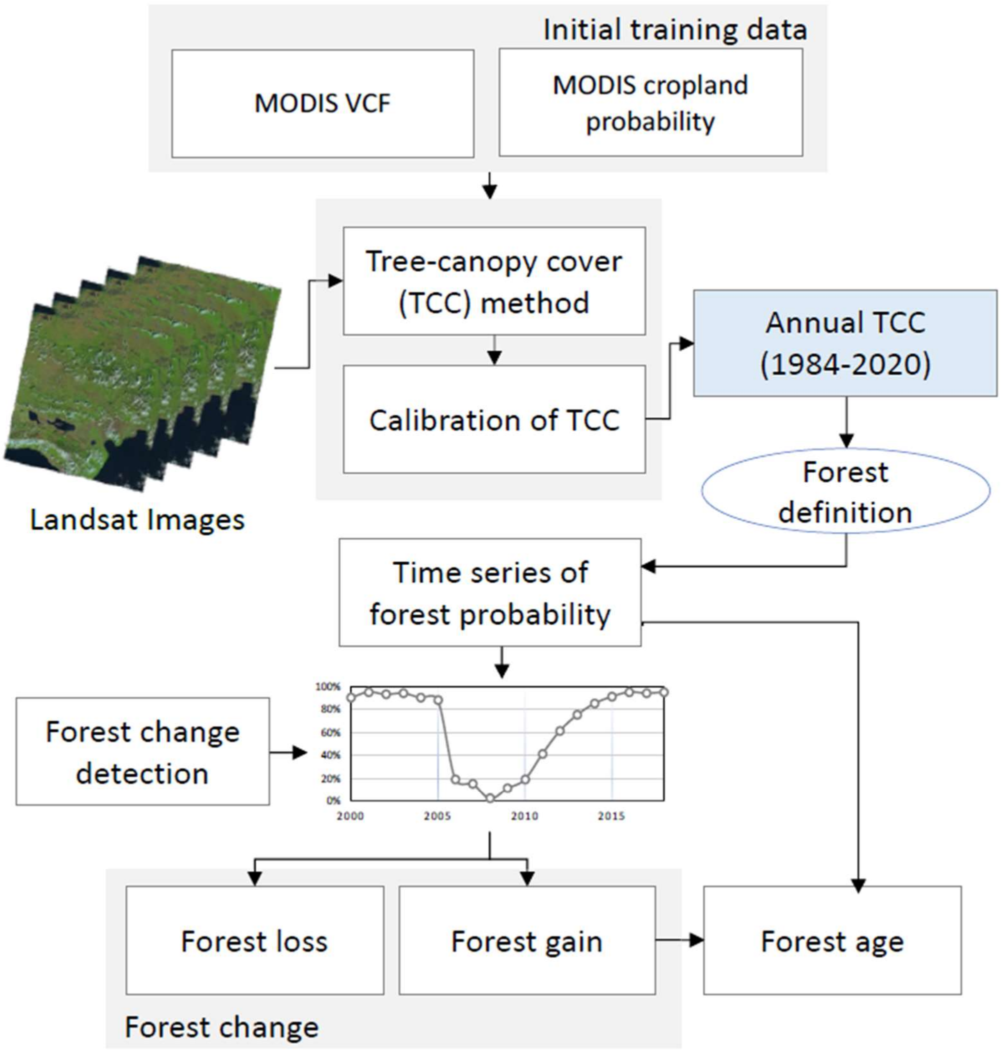

Supplementary Figure 18. Process for estimating tree-canopy cover (TCC) and forest probability, change and age.

Supplementary Table 1.

Formulation of the various growth models tested:

(1)	$w(x) = a + b * \log(x)$		Assman 1943; Prodan 1951; Schmidt 1967
(2)	$w(x) = a * (1 - \exp(-b * x))^{c}$	Chapman-Richards polymorphic	Pretzsch2009, Perin2013
(3)	$w(x) = b * (1 - \exp\left(\frac{a-x}{c}\right))$	Mitscherlich	Perin2013
(4)	$w(x) = b * exp(-\exp\left(\frac{a-x}{c}\right))$	Gompertz	Perin2013
(5)	$w(x) = \exp(a + b * (\frac{1}{x})^{c})$	Baily and Clutter polymorphic	Perin2013
(6)	$w(x) = b/(1 + (\frac{x}{a})^{-c})$	log-logistic	Perin2013
(7)	$w(x) = \left(\frac{b}{\pi}\right) * (\frac{\pi}{2} + \operatorname{atan}\left(\frac{x-a}{c}\right))$	Arc tangent	Perin2013
(8)	$w(x) = a * (1 - \exp(-b * x)) + c$	Negative exponential	Fekedulegn1999, Philip1994
(9)	$w(x) = (a * x + b) * (1 - \exp(-(\frac{x}{c})^{d})$	Duplat and Tran-Ha 1	Perin2013
(10)	$w(x) = a * (1 - b * \exp(-c * x)) + d$	Monomolecular	Fekedulegn1999, Drapper and Smith 1981
(11)	$w(x) = (a * \log(x) + b) * (1 - \exp\left(-(\frac{x}{c})^{d}\right))$	Duplat and Tran-Ha 4	Perin2013
(12)	$w(x) = (a^{1-b} - c * \exp(-d * x))^{\frac{1}{1-d}} + f$	von Bertalanffy	Fededulegun1999, Bertalanffy1957
(13)	$w(x) = (a * x + b) * \left(1 - \exp\left(-(\frac{x}{c})^{d}\right)\right) + f * x$	Duplat and Tran-Ha 2	Perin2013
(14)	$w(x) = (a * \log(x) + b) * \left(1 - \exp\left(-(\frac{x}{c})^{d}\right)\right) + f * x$	Duplat and Tan-Ha 5	Perin2013
(15)	$w(x) = (a * x + b) * \left(1 - \exp\left(-(\frac{x}{c})^{d}\right)\right)^{g} + f * x$	Duplat and Tran-Ha 3	Perin2013

Equations ordered by the number of coefficient shape parameters.
