# Supplementary material for: Russian forests show strong potential for young forest growth
Source: Commun Earth Environ. 2025 Jan 30;6(1):71. doi: 10.1038/s43247-025-02006-9 (PMC11782080; doi:10.1038/s43247-025-02006-9)
Supplement: Supplementary file 2 — Reporting Summary [file 43247_2025_2006_MOESM2_ESM.pdf]

nature portfolio

Corresponding author(s): *Double-blind peer review submissions: write DBPR and your manuscript number here instead of author names.*

Last updated by author(s): *YYYY-MM-DD*

Reporting Summary

Nature Portfolio wishes to improve the reproducibility of the work that we publish. This form provides structure for consistency and transparency in reporting. For further information on Nature Portfolio policies, see our Editorial Policies and the Editorial Policy Checklist.

Statistics

For all statistical analyses, confirm that the following items are present in the figure legend, table legend, main text, or Methods section.

n/a	Confirmed	
☐	☒	The exact sample size (*n*) for each experimental group/condition, given as a discrete number and unit of measurement
☐	☒	A statement on whether measurements were taken from distinct samples or whether the same sample was measured repeatedly
☐	☒	The statistical test(s) used AND whether they are one- or two-sided<br>*Only common tests should be described solely by name; describe more complex techniques in the Methods section.*
☐	☒	A description of all covariates tested
☐	☒	A description of any assumptions or corrections, such as tests of normality and adjustment for multiple comparisons
☐	☒	A full description of the statistical parameters including central tendency (e.g. means) or other basic estimates (e.g. regression coefficient) AND variation (e.g. standard deviation) or associated estimates of uncertainty (e.g. confidence intervals)
☒	☐	For null hypothesis testing, the test statistic (e.g. *F*, *t*, *r*) with confidence intervals, effect sizes, degrees of freedom and *P* value noted<br>*Give P values as exact values whenever suitable.*
☒	☐	For Bayesian analysis, information on the choice of priors and Markov chain Monte Carlo settings
☒	☐	For hierarchical and complex designs, identification of the appropriate level for tests and full reporting of outcomes
☒	☐	Estimates of effect sizes (e.g. Cohen's *d*, Pearson's *r*), indicating how they were calculated

Our web collection on statistics for biologists contains articles on many of the points above.

Software and code

Policy information about availability of computer code

Data collection	Forest age was calculated based on 30-meter, annual-resolution estimates of tree cover spanning the boreal region from 1984 to 2020 derived from Landsat collection-1 surface reflectance images (http://landsat.usgs.gov). Stand-age data were combined with ICESat-2 forest height samples in 20 m ? 11 along-track segments with non-linear forest growth models to estimate the lands capacity to grow trees and predict where vertical growth gaps exist. We assembled vegetation heights from the National Aeronautical and Space Administration's (NASA) Ice Cloud and Elevation Satellite-2 (ICESat-2) Advanced Topographic Laser Altimeter (ATLAS)-ATL08 height of canopy 20 m segments (hcan, 98% height profile) version 5 dataset77 from the National Snow and Ice Data Center78.
Data analysis	Forest height is correlated to woody biomass and carbon storage and modeling height growth as a function of time enables prediction of above ground biomass potential. In this study, we had three distinct steps:<br>1) Build a database of spatially and temporally coincident forest height and age observations;<br>2) Map expected forest height across the region by fitting a range of forest growth models relating stand height to age and mapping the predictions across the boreal domain; and<br>3) Detect growth gaps by subtracting actual from expected forest height across the region.

For manuscripts utilizing custom algorithms or software that are central to the research but not yet described in published literature, software must be made available to editors and reviewers. We strongly encourage code deposition in a community repository (e.g. GitHub). See the Nature Portfolio guidelines for submitting code & software for further information.

Data

Policy information about availability of data

All manuscripts must include a data availability statement. This statement should provide the following information, where applicable:

- Accession codes, unique identifiers, or web links for publicly available datasets
- A description of any restrictions on data availability
- For clinical datasets or third party data, please ensure that the statement adheres to our policy

Data Availability Statement
All data and materials used in the analyses are available on Github. https://github.com/mwooten3/ZonalStats-3DSI/tree/main/data
Code Availability Statement
All data and materials used in the analyses are available on Github. https://github.com/mwooten3/ZonalStats-3DSI/tree/main/data

Human research participants

Policy information about studies involving human research participants and Sex and Gender in Research.

Reporting on sex and gender	N/A
Population characteristics	*Describe the covariate-relevant population characteristics of the human research participants (e.g. age, genotypic information, past and current diagnosis and treatment categories). If you filled out the behavioural & social sciences study design questions and have nothing to add here, write "See above."*
Recruitment	*Describe how participants were recruited. Outline any potential self-selection bias or other biases that may be present and how these are likely to impact results.*
Ethics oversight	*Identify the organization(s) that approved the study protocol.*

Note that full information on the approval of the study protocol must also be provided in the manuscript.

Field-specific reporting

Please select the one below that is the best fit for your research. If you are not sure, read the appropriate sections before making your selection.

☐ Life sciences ☐ Behavioural & social sciences ☒ Ecological, evolutionary & environmental sciences

For a reference copy of the document with all sections, see nature.com/documents/nr-reporting-summary-flat.pdf

Ecological, evolutionary & environmental sciences study design

All studies must disclose on these points even when the disclosure is negative.

Study description	Stand-age data were combined with ICESat-2 forest height samples in 20 m ? 11 along-track segments with non-linear forest growth models to estimate the lands capacity to grow trees and predict where vertical growth gaps exist
Research sample	We estimated growth rate patterns by collating coincident measurements of forest height and age. The locations of the point-based h_canopy_20 m observations, representing the 20 m x 11 m ATL08 segment centroids, provided the spatial index for extracting coincident stand age values. All quality filtered ATL08 observations corresponding with forested pixels were retained, resulting in 45,347,339 segments across the boreal domain. 39,259,745 forest age observations were older than the 36-year Landsat record, and 6,087,594 segments with coincident age estimates were available for analysis.
Sampling strategy	All available data were included within the study domain.
Data collection	Data were derived from satellite sensors.
Timing and spatial scale	1984-2020 circumboreal forest.
Data exclusions	We selected 1 of 15 forest growth models per tile using root mean square errors (RMSEs) and sum squared errors (SSEs) to evaluate model performance. Models with RMSEs > 6, SSEs > 250, and curves predicting height in year 50 (i.e., SI50) 50 < 2 or > 40 m were excluded from analysis. Of the 15 models applied, those with the lowest RMSE and SSE were selected as output.
Reproducibility	We experimented with reproducing results at different geographical scales from 0.1 to 1 degree.
Randomization	Not relevant to our analysis.

Blinding	Not relevant to our remote sensing analysis.

Did the study involve field work? ☐ Yes ☒ No

Reporting for specific materials, systems and methods

We require information from authors about some types of materials, experimental systems and methods used in many studies. Here, indicate whether each material, system or method listed is relevant to your study. If you are not sure if a list item applies to your research, read the appropriate section before selecting a response.

Materials & experimental systems

n/a	Involved in the study
☒	☐ Antibodies
☒	☐ Eukaryotic cell lines
☒	☐ Palaeontology and archaeology
☒	☐ Animals and other organisms
☒	☐ Clinical data
☒	☐ Dual use research of concern

Methods

n/a	Involved in the study
☒	☐ ChIP-seq
☒	☐ Flow cytometry
☒	☐ MRI-based neuroimaging
